# Non and Epigenetic Mechanisms in Regulation of Adaptive Thermogenesis in Skeletal Muscle

**DOI:** 10.3389/fendo.2019.00517

**Published:** 2019-08-13

**Authors:** Bijayashree Sahu, Sunil Pani, Gourabamani Swalsingh, Naresh C. Bal

**Affiliations:** School of Biotechnology, KIIT University, Bhubaneswar, India

**Keywords:** skeletal muscle, muscle mitochondria, calcium handling mechanisms, SERCA (sarco(endo)plasmic reticulum Calcium ATPase), energy homeostasis, thermogenesis

## Introduction

Homeothermic mammals including humans produce heat (also termed thermogenesis) inside their body to maintain constant body temperature. It has been shown that thermogenesis is elevated by several external factors; prominent being cold, diet, and physical exercise. This modulation ability is termed as “Adaptive Thermogenesis (AT).” Intake of high calorie diet was also shown to increase thermogenesis in laboratory animals, a phenomenon termed as diet-induced thermogenesis (DIT) (1). Research on finding out the mechanisms of DIT intensified in the recent years, as obesity and associated metabolic disorder increased rapidly all over the world. It is hoped that mechanisms of AT can be targeted to increase energy expenditure and provide protection against metabolic diseases including obesity. Brown adipose tissue (BAT) and skeletal muscle have emerged as the two major sites of AT. Major heat producer in BAT is a protein called uncoupling protein (UCP) 1 that dissipates proton gradient in mitochondria and thereby resulting in heat production (2). Few additional thermogenic mechanisms have also been reported in BAT such as futile TG lipolysis/esterification, creatine/phosphocreatine cycling, and ATP-dependent Ca^2+^-cycling (3–5). The understanding of mechanisms of AT in the skeletal muscle has been slow and forms the major focus of this review.

## Potential Contributors of AT in the Skeletal Muscle

In the skeletal muscle, contributors to AT can be proteins of (i) Calcium (Ca^2+^)-handling, (ii) contractile apparatus, and (iii) mitochondrial metabolism. ATP utilization by these proteins can vary due to their relative expression and post-translational modification. This is additionally regulated by the abundance of signaling molecules (ions, lipids, etc.) in the micromilleu which in turn is determined by their transporter proteins. The term epigenetic has been classically applied to describe changes in protein expression via differential chromosomal compaction without genetic alteration. Herein, a more liberal interpretation of “epigenetics” is taken that encompasses all functional alterations irrespective of inheritance and genetic modifications. Such changes include transcriptional, post-transcriptional, translational, post-translational. We additionally discuss about the modulation of AT in the skeletal muscle by factors like microRNA, vitamins, and hormones.

## Calcium Signaling and Epi-genetics in Skeletal Muscle

Recent discoveries have made it clear that Ca^2+^-handling proteins are the key mediators of AT in the skeletal muscle (6). Epigenetic modifications of these proteins alter their physiological function thereby influence whole body metabolism. Ca^2+^ signaling proteins involved in different aspects of muscle function are: Ca^2+^-release from Sarcoplasmic reticulum SR (Ryanodine Receptor (RyR), IP3R); Ca^2+^-buffering inside SR (calsequestrin, calreticulin, parvalbumin, and sarcalumenin); Ca^2+^-uptake into SR (SERCA, SLN, PLB, and MLN); Ca^2+^-transport across the membrane (PMCA, MCU, MICU, TRP channels, STIM, Orai1). Function of these proteins can be regulated by epigenetic mechanisms and contribute to adaptive thermogenesis.

## SERCA Determines Intramyocellular Calcium Dynamics

SERCA has emerged as the primary determinant of ATP utilization in muscle (7–9), therefore is a key player in adaptive thermogenesis. Mammals possess three distinct SERCA isoforms: SERCA 1 is predominantly expressed in fast twitch fibers; SERCA 2 is expressed in cardiac and slow twitch skeletal muscles; and SERCA 3 is found mostly in non-muscle cells (8, 10). SERCA function is chiefly regulated by micropeptides and post-translational modifications via factors including hormones (11).

### Regulation of SERCA Activity by Micropeptides

**Figure 1:**
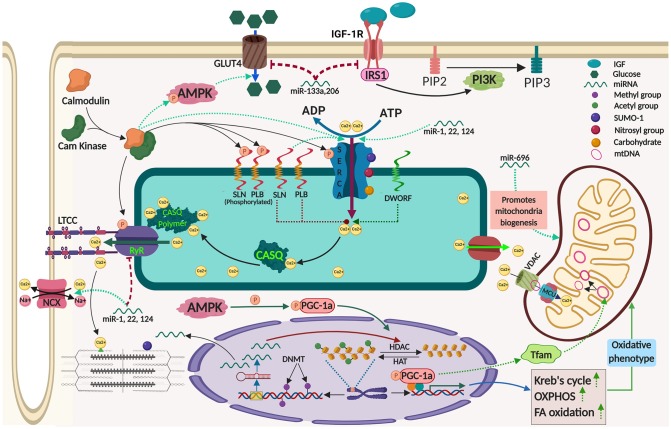
Mechanisms contributing to adaptive thermogenesis in the skeletal muscle and its regulation by epigenetic processes. The image was created by Biorender.com.

Among the micropeptide regulators of SERCA, phospholamban (PLB) is the best characterized. PLB binds only to Ca^2+^ free state of SERCA and acts as an inhibitor. Once Ca^2+^ concentration in the microenvironment increases it gets dislodged from the SERCA allowing its unhindered activity (12). In contrast, SLN can interact with Ca^2+^ bound confirmations of SERCA promoting futile Ca^2+^ transport thus enhancing higher ATP utilization (10, 13, 14). This function of SLN leads to elevated energy expenditure resulting in heat production in the muscle and serves as a major regulator of non-shivering thermogenesis (NST). Interestingly, SLN is predominantly found in atria and skeletal muscle, while PLB expression is restricted to ventricle. PLB inhibition of SERCA is regulated by its phosphorylation at Ser-16 and Thr-17 residues by Ca^2+^/calmodulin- dependent protein kinase (CaM kinase) and protein kinase-A, respectively. On the other hand, SLN function is regulated by several factors; like its differential expression, its phosphorylation at Thr-5 residue, ATP concentration and membrane lipid composition (15–17). Recent studies have identified few more micropeptides like DWORF, MLN, and Ankyrin1 as regulators of SERCA (18–20). Although the exact function of these new regulators is not fully understood, their contribution to AT of muscle has been speculated. Interestingly, DWORF acts differently from all the other micropeptide regulators, even though is conserved and binds to the same SERCA groove as PLB and SLN. However, the expression of DWORF is limited to heart and slow twitch fibers like soleus, and it is proposed to be an activator of SERCA function (18). The fine tuning of SERCA activity by these micropeptide regulators might have a big impact on whole body energy homeostasis (shown in Figure 1), and an unrealized potential in adaptive thermogenesis.

### Regulation of SERCA Activity by Post-translational Modification

Several post-translational modifications including glutathionylation, SUMOylation, O-GlcNAcylation, glycosylation, nitration, and acetylation have been proposed to modulate SERCA activity. Glutathionylation of SERCA mainly occurs at Cys674 residue and has isoform specific effects (11, 21, 22). Peroxinitrite, one critical molecule in glutathionylation has different effects on distinct SERCA isoforms. Peroxynitrite promotes SERCA2a glutathionylation increasing Ca^2+^-transport during artherosclerosis whereas, it inhibits Ca^2+^-uptake of SERCA1a by oxidizing it at lower levels of glutathione (21, 22). Several investigations addressed SUMOylation of SERCA, although SERCA2a is better studied. In this type of modification small ubiquitin like modifier (SUMO) 1 binds to SERCA at Lys 480 & 585 residues (11, 23). SUMOylation upregulates SERCA2a activity and is cardioprotective, while its effects on SERCA1a are still unknown. Literatures suggested SUMOylation-mediated increased SERCA function is due to blocking of acetylation of SERCA (11). Acetylation is another post-translational modification that acts antagonistic to that of SUMOylation (24). Glycosylation involves addition of carbohydrate molecules to SERCA at elevated glucose levels in the muscle hindering SERCA2a activity and therefore has been considered as a pharmacological target in diabetes and cardiovascular diseases (25). O-GlcNAcylation is one of the specific modification among glycosylation in which N-acetylglucosamine is added to SERCA2a at serine and threonine residues. Similar to other glycosylation. O-GlcNAcylation reduces SERCA2a activity directly as well as indirectly by increasing PLB expression level during hyperglycemia (25). Another post-translational modification of SERCA is nitration, where a nitro group is added to tyrosine residues. Level of nitration in SERCA2a increases during hyperglycemic condition as a result of higher oxidative stress (26), hence might be critical in metabolic diseases like diabetes. Although regulation of SERCA activity by various epigenetic and post-translational modifications are important in energy homeostasis but their individual roles in AT are yet to be deciphered.

## RyR

RyR is the primary Ca^2+^-release channel of SR; while RyR1 is dominant in skeletal muscle, RyR2 predominates in cardiac. Genetic alterations of RyR that make the channel leaky are the basis of diseases like malignant hyperthermia and central core disease. Some studies have demonstrated epigenetic regulation of RyR gene expression in health and disease, suggesting that same regulations might play important role in adaptive thermogenesis. Rokach et al. showed that in recessive RYR1-linked myopathies, Histone deacetylase (HDAC4 and HDAC5), and DNA methyltransferases (DNMT1 and DNMT2) are upregulated leading to chromatin condensation and reduction in RyR1 transcript level. Increased HDACs also sequester mef2, a major muscle transcriptional factor that might affect expression of many genes including miRNAs. They have proposed that these epigenetic regulators can be targeted pharmacologically to treat several inherited muscular disorders (27). Thiol rich RyR protein is responsive toward phosphorylation, oxidation, nitrosylation, and glutathionylation (28–30). RyR1 phosphorylation at serine and threonine residues by CamKII and PKA, respectively, has been suggested to make the channel unstable and cause leak of Ca^2+^ from the SR having severe consequences on Ca^2+^-signaling in the cytosol. RyR1 phosphorylation plays a key role in adaptation to cold (31). Nitrosylation of RyR at thiol groups is facilitated by O_2_ level in the muscle and increases Ca^2+^-release from the channel (29). Thus, RyR might play an important role in AT either directly by Ca^2+^-induced bioenergetics or indirectly via Ca^2+^-mediated signaling.

## Epigenetic Mechanisms Regulate Energetics of Contractile Apparatus

AT of skeletal muscle is highly dependent on ATP utilization by the contractile apparatus. This can be regulated by relative myosin isoform expression, their post-translational modifications and bioenergetics of contractile apparatus. Interestingly, all are affected during physical activity for AT recruitment in the muscle. Exercise mediates conversion of slow twitch-to-fast twitch fiber in soleus by myosin heavy chain (MHC) gene expression via chromatin remodeling. During this transition, histone deacetylation down-regulates the MHC-I gene, whereas histone acetylation upregulate fast type IIx and IIb MHC genes. Alteration in relative expression of myosin isoforms has also been seen in condition that influence AT such as aging, high fat/sugar diet; whereas in cold adaptation it is still less explored. Interestingly, a newly characterized confirmation of myosin called super relaxed state (SRX) by Stewart et al. throws light on its potential role in cold adaptation (32). In normal condition ATP turnover by myosin is <0.1 s, but in SRX, myosin can slowly split ATP with turnover time (~230 s at 24°C) inside the core myosin filament in skeletal muscle. Further, activity of SRX myosin can be enhanced in cold through phosphorylation by Myosin light chain kinase (MLCK) or by substitution of GTP for ATP.

Energy used by myosin is also influenced by epigenetic modifications of other partner proteins of contractile apparatus. Troponin-T binds to tropomyosin and control acto-myosin cross bridge formation thereby determining ATP usage by myosin. Sumoylation of actin by SUMO-1 has been reported in the skeletal muscle in response to exercise training indicating its potential in adaptive thermogenesis (33). Resistance exercise training in older adults showed marked increase in the expression of slow troponin-T isoforms by alternative splicing of the pre-mRNA. Parallely differential expression of several tropomyosin isoforms (Tpm1.6, Tpm1.7, Tpm2.1, etc.) distinctly regulate myosin ATPase thus affecting energy expenditure and thermogenesis by the muscle (34). Hence, epigenetic regulation of contractile machinery proteins may serve as a mechanism to influence energy usage in the muscle having an effect on adaptive thermogenesis.

## Role of AT in Modulation of Mitochondrial Metabolism

The SR Ca^2+^-handling machinery and contractile apparatus determine the cytosolic Ca^2+^-level that can regulate the Ca^2+^-signaling into the nucleus and mitochondria. Thus, all the physiological functions of the skeletal muscle is intimately coupled to mitochondrial metabolism, which in turn depend on modulation of: (1) mitochondrial transcriptional machinery, (2) nuclear gene expression, and (3) Ca^2+^-influx into the mitochondrial lumen. Each of these components can be affected by epigenetic mechanisms mediating the adaptive changes in the thermogenic state of the skeletal muscle. Mitochondrial metabolism is critically governed by PGC-1α, a master transcriptional regulator encoded by a nuclear gene (PPARGC1A). The activity of PGC-1α in skeletal muscle regulates chromatin remodeling via by acetylation/deacetylation (SIRT1, GCN5) and methylation (PRMT1) in response to energetically drained energy/substrate conditions like fasting, exercise, calorie restriction (35, 36). The PGC-1α function is fine-tuned by post-translational modifications, including phosphorylation (by AKT, GSK-3, CLK2, CAMKKB, PKA, S6K, P38, and AMPK), dephosphorylation (calcineurin), ubiquitination (SCF), and O-GlcNAc modification. Level of PGC-1α protein and its activity is closely intertwined with muscle metabolic status via epigenetic mechanisms that respond to number of physiological cues like hypoxia, exercise, calorie restriction, and aging.

AMPK serves as another key energy sensor and its activation enhances glucose uptake (by increasing GLUT4 translocation), fatty acid oxidation, and mitochondrial biogenesis in the skeletal muscle. In fact, AMPK can also regulate PGC-1α by direct phosphorylation at Thr177 and Ser538 residues thereby increasing the transcription of PGC-1α-target genes (37). Interestingly, AMPK is activated by high AMP/ATP ratio which is usually associated with elevated cytosolic Ca^2+^-level in the muscle, where Ca^2+^-dependent signaling is already induced. Therefore, AMPK serves as a key integrator of Ca^2+^-dependent and ATP-dependent pathways.

Activation of mitochondrial function in the skeletal muscle is also influenced by Ca^2+^-regulated transcription factors like (CaMK) isoforms and calcineurin/NFAT. Role of calcineurin/NFAT in muscle fiber type determination is well-known (38), but recent studies have shown that they decode the local Ca^2+^-signal and mediate that to nucleus for expression of mitochondrial metabolic genes (39). Another important mitochondrial transcriptional regulator in the muscle is myocyte enhancer factor (MEF) 2. It is suggested that MEF-2 strikes a balance between HDACs-mediated transcriptional repression and activation by factors like NFAT, PGC-1α, and MAPK.

The acute increase in oxidative metabolism in muscle is achieved by Ca^2+^-influx into the mitochondria and the same process can also be tapped for adaptive thermogenesis. Mitochondrial Ca^2+^-influx is governed by two channels: voltage-dependent anion channel (VDAC), located on the outer mitochondrial membrane and mitochondrial calcium uniporter (MCU) on the inner mitochondrial membrane. It has been shown that MCU overexpression results in increased protein synthesis leads to hypertrophic phenotype and protects mice from denervation-induced atrophy in muscles via IGF1/AKT and PGC-1α4 pathways. Important role of MCU in skeletal muscle metabolism is further highlighted by the identification of mutations in MICU1, an MCU regulator, in patients with proximal muscle weakness (40). On the other hand, VDAC1 expression was significantly up-regulated in the skeletal muscle upon cold adaptation of mice which suggest role for VDAC in adaptive thermogenesis (41). Recent studies has also thrown light on Mitochondrial Ca^2+^-influx due to SR-Ca^2+^-handling by the action of a small protein called SLN that regulate SERCA-mediated Ca^2+^-uptake. Evidences shows that mitochondrial metabolism is intertwined with muscle thermogenic genes speculating its potential role in AT.

## Other Factors in AT in Muscle

AT can further be modulated by additional factors such as miRNA, vitamins, and hormones. We chose to discuss them separately as the role of these factors is broad and their role in skeletal muscle AT is being updated every day.

### MicroRNAs

The miRNAs are short non-coding RNA of 20-24 nucleotides that modify target gene expression post-transcriptionally. Several miRNAs have been discovered and characterized in the skeletal muscle termed as myo-miRs (Table 1). Research has evidenced that physiological responses like lipid utilization, endothelial function and mitochondrial activity are modulated by miRNAs in skeletal muscle. Expression of NCX (sodium/calcium exchanger 1), inositol-1,4,5-trisphosphate receptor 1 (IP3R1), and SERCA-2a (sarcoplasmic reticulum Ca^+2^ ATPase-2a) are targeted by miR-1, miR-25, and miR-214, respectively. A recent study showed that miR-1a, miR-22, and miR-124 cause RyR1 down-regulation in the skeletal muscles of multi-minicore disease. Interestingly, miRNA has been shown to have additional role other than mRNA regulation by directly interfering with protein-protein interaction. Soller et al. have shown that miR-1 and miR-21 binds to PLB with low dissociation constant reversing its inhibition on SERCA (43). High-fat diet (HFD) induced modifications of gene expression is in part mediated by miRNAs such as miR-1a, miR-133a, and miR-206. Both miR-133a and miR-206 upregulated after HFD intake targeting insulin-growth factor (IGF)-1 and IGF-1R mRNA leading to drop in the IGF-1 signaling pathway (59).

**Table 1:** MicroRNA-mediated regulation of protein expression in skeletal muscle.

**miRNAs**	**Expression**	**Target proteins**	**Physiological alteration**	**Experimental setup**	**References**
miR-449a	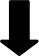	HDAC	Diabetic skeletal muscle	HDAC inhibition using suberoylanilide hydroxamic acid (SAHA)	(42)
miR-22	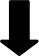	RYR, HDAC-4, 5; SERCA-2A	Methylation of RYR and impared calcium homeostasis; decrease in cardiac expression levels for SERCA-2A	Over-expressing HDAC-4 and HDAC-5 or knocking down ryr1 by siRNA silencing;miR-22^−/−^ Mice, calcineurin transgenic (CnA-Tg) mice and pressure overload by transverse aortic constriction (TAC)	(43, 44)
miR-7a, miR-8519	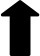	PxRYR	High Ca^2+^ release from SR in *Plutella xylostella* (L.)	PxRyR knockout	(45)
miR-1, miR-206, miR-23a, miR-133a	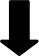	IGF-1, IGF-1R; PAX3, HDAC4	Hypertrophy; angiogenesis, actin filament assembly	Acute resistance exercise and chronic resistance exercise, 12 weeks, functional overload and cyclic exercise; MZdicer ^+miR−430^ and mono-miRNA-KO	(46–50)
miR-194	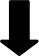	AKT, GSK3β, OXPHOS	Insulin signaling, glucose uptake, oxidative phosphorylation	miRNA knockdown strategy in high fat fed rat	(51)
miR-696, miR-761	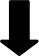	Pgc-1α	Metabolism, mitochondrial biogenesis	Chronic treadmill running, 4 weeks	(52, 53)
miR-106b, miR-27a and miR-30d; mir-17	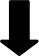	GLUT4, MAPK-14 and PI3K	Glucose catabolism and glucose uptake	(MTg-AMO) antisense oligonucleotides technology and miRNA knockdown strategy; knockdown of endogenous miR-17	(54)
miR-696, miR-494,	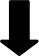	(mtTFA) & (Foxj3); NRF-1, Pgc-1α,	Mitochondrial biogenesis	Acute swimming; voluntary wheel, 8 weeks	(55, 56)
miR-126	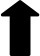	VEGF, VEGF-R2, ACE2, PI3-K2	Hypertrophy, vascularization	Chronic swimming, 10 weeks	(57, 58)
miR-16, miR-21	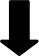				
miR-1 miR-21	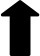	SERCA, PLB	Calcium signaling	Purification and assay of SERCA and PLB from rabbit skeletal muscle	(43)

Role of miRNA in various types of exercise training has been studied in details (Table 1). Exercise training (both acute and endurance) down-regulate and upregulate different sets of miRNA that positively co-regulate the expression of HDAC4 and NRF-1 leading to muscle regeneration, and mitochondrial biogenesis (60). Angiogenesis via VEGF-A expression are regulated by opposing action of miR-206 and miR-1 (61). The miR-1 suppress HDAC4, in turn, upregulates follistatin (FLN), a fusion promoting factor and antagonize the myogenic inhibitor myostatin (MSN) and SARS (seryl-tRNA synthetase) contrary to the action of miR-206 (62). It has been studied that muscle hypertrophy was enhanced by expression of c-Met, HGF, IGF-1, SRF, and LIF genes by down-regulation of miR-1 and miR-133a via functional overload in plantaris muscle (49). Exercise upregulated PGC-1α mediates its effects by downregulating miR-696 and upregulating miR-23 expression leading to mitochondrial biogenesis in skeletal muscle (52, 63). Two miRNAs miR-499 and miR-208b coexpressed with Myh7 and Myh7b genes in slow myofiber genes and regulated by miR-208a (expressed in Myh6 gene) for slow to fast twitch fiber type switching (64). Thyroid hormone upregulated miR-133a1 in a Thyroid receptor (TR)-dependent manner and promotes slow-to-fast muscle switch by repressing TEA domain family member 1 (TEAD1) (65). Expression of the phosphatase and tensin homolog (PTEN) and forkhead box O1 (FOXO1) is suppressed by miR-486 to promote muscle hypertrophy (66).

### Hormones and Vitamins

Several hormones influencing muscle metabolism (like insulin, leptin, GLP-1, thyroid) undergo drastic changes in their circulating levels in conditions that can have impact on AT (67). Steroids and thyroids are the best studied hormones to regulate protein expression in muscles and catecholamines are known to enhance muscle energy expenditure (68). Similarly, many vitamins (e.g., Vitamin D) directly or indirectly mediate their effects by altering intracellular calcium dynamics. Their conspicuous pleiotropic gene expression are known to modulate energy status and also affect protein expression in different muscle types (69). However, their specific role in regulating AT in the skeletal muscle is still poorly defined. It is interesting to note that few cytokines that were previously thought not to regulate energy homeostasis have been demonstrated to do so and are now reclassified as hormones. Defining their function in regulation of muscle energy utilization will clarify their role in AT.

## Concluding Remarks

Epigenetic mechanisms significantly control AT in skeletal muscle. Skeletal muscle constitute more than 40% of the body weight and its energy status serves as a major determinant of metabolic rate. Even a minute alteration in muscle metabolic state can have remarkable change in the whole body energy expenditure. So, epigenetic mechanisms regulating skeletal muscle AT can hugely control energy homeostasis. Some of these mechanisms can serve as good targets for manipulation of energy expenditure to counter metabolic disorders such as obesity and type 2 diabetes. Researchers have tried to develop pharmacological agents to tap muscle metabolism for treatment of obesity. However, these efforts have not resulted in any product yet as epigenetic control of muscle AT is still not well-defined. Another major direction that future studies should address is whether epigenetic regulation of muscle AT affects the functioning of other organs. Secretion of myokines and their effect in modulation of many organs including white fat depot, liver has recently been illustrated. Another poorly defined area is role of vitamins in regulation of muscle metabolism and NST capacity that can play a key role in adaptive thermogenesis. Future studies in these directions will unravel mechanisms of epigenetic regulation that will help in designing strategies to counter metabolic disorders by activation of AT.

## Author Contributions

SP prepared the figure and BS prepared the table. All authors drafted the manuscript together, critically revised the work, and approved the final version.

### Conflict of Interest Statement

The authors declare that the research was conducted in the absence of any commercial or financial relationships that could be construed as a potential conflict of interest.
